# ‘Dose-to-Mother’ Deuterium Oxide Dilution Technique: An Accurate Strategy to Measure Vitamin A Intake in Breastfed Infants

**DOI:** 10.3390/nu9020169

**Published:** 2017-02-21

**Authors:** Veronica Lopez-Teros, Ana Teresa Limon-Miro, Humberto Astiazaran-Garcia, Sherry A. Tanumihardjo, Orlando Tortoledo-Ortiz, Mauro E. Valencia

**Affiliations:** 1 Nutritional Sciences, Department of Chemical and Biological Sciences, University of Sonora, Hermosillo, Sonora 83000, Mexico; mauro@ciad.mx; 2 Department of Nutrition, Research Center for Food and Development (CIAD, AC), Hermosillo, Sonora 83304, Mexico; analimonmiro@gmail.com (A.T.L.-M.); hastiazaran@ciad.mx (H.A.-G.); otortoledo@ciad.mx (O.T.-O.); 3 Department of Nutritional Sciences, University of Wisconsin-Madison, Madison, WI 53706, USA; sherry@nutrisci.wisc.edu

**Keywords:** deuterium oxide dilution, infants’ vitamin A adequacy, breast milk retinol, breast milk carotenoids, breast milk intake, VA status in lactating women

## Abstract

In Mexico, infants (0–2 years old) show the highest prevalence of vitamin A deficiency (VAD), measured by serum retinol concentrations. Thus, we consider that low vitamin A (VA) intake through breast milk (BM) combined with poor weaning practices are the main factors that contribute to VAD in this group. We combined the assessment of VA status in lactating women using BM retinol and a stable isotope ‘dose-to-mother’ technique to measure BM production in women from urban and agricultural areas. Infants’ mean BM intake was 758 ± 185 mL, and no difference was observed between both areas (*p* = 0.067). Mean BM retinol concentration was 1.09 μmol/L, which was significantly lower for the agricultural area (*p* = 0.028). Based on BM retinol concentration, 57% of women were VAD; although this prevalence fell to 16% when based on fat content. Regardless of the VA biomarker used here, infants from the urban and agricultural areas cover only 66% and 49% of their dietary adequate intake from BM, respectively (*p* = 0.054). Our data indicate that VAD is still a public health concern in Mexico. Adopting both methods to assess VA transfer from the mother to the breastfed child offers an innovative approach towards the nutritional assessment of vulnerable groups.

## 1. Introduction

Vitamin A deficiency (VAD) is recognized by the World Health Organization (WHO) as one of the main nutritional problems that affects developing countries, where children under 5 years, and pregnant and lactating women are the most vulnerable groups [1]. In Mexico, VAD is a severe subclinical public health problem, particularly for children <5 years old; unfortunately, there is not much information on the VA status of Mexican pregnant and lactating women.

A study performed in lactating women from northwest Mexico (Sonora) [2], showed that more than 50% had VAD using serum retinol concentrations as an indicator, which is indicative of a severe public health problem [1]. Additionally, the mean breast milk (BM) retinol concentration was 1.18 µmol/L, which is close to 1 µmol/L observed in regions where VAD is common [3]. This VA concentration in breast milk is barely enough to satisfy the metabolic needs of the breastfed child, leaving little opportunity to accumulate VA reserves in the liver for times of low intake [4].

The Mexican National Nutrition Survey 2012 [5], shows that VAD is of concern in children <2 years old. This suggests that low intake of VA through BM combined with poor weaning practices could be the main factors contributing to VAD in this age group.

Efforts to reduce VAD in children under 5 years have been carried out for over 20 years in Mexico. Preformed VA supplements are administered to children 6 months to 4 years old (60 mg; 210 µmol retinyl palmitate) during national immunization campaigns. Although there is an immediate improvement in VA status, its effect is limited in time (~3 months), whereby circulating VA returns to pre-dose concentrations and VAD reappears in areas with low VA intakes [6]. Additionally, recent estimates in a sample of preschoolers from northwest Mexico, showed that 48% had low serum retinol concentrations [7].

Given the above, to adequately fight VAD in breastfed infants, it is essential that the mother’s VA status be optimal in order to fulfill the infant's metabolic needs and be sufficient to maintain VA stores and prevent VAD [4]. In lactating women, BM retinol concentration is a unique indicator related to the mother’s VA status, is sensitive to dietary VA intake [8,9], and may predict the breastfed infant’s VA status [10,11]. Furthermore, BM retinol may not be affected by systemic inflammation [12], although this requires more research considering that serum retinol bound to retinol-binding protein contributes to BM retinol [13] and is decreased during inflammation [14].

Even though BM has a central role in energy and nutrient intake, which are essential for the infant’s growth and development, only limited records document BM intake [15]. Furthermore, the only available information associating BM intake and VAD was performed in Senegalese breastfed infants showing that VA intake was close to their recommended dietary adequate intake [16].

The gold standard to measure BM intake in humans is the ‘Dose-to-mother’ deuterium oxide dilution (DOD) technique [17,18], which measures the turnover rate of body water after an oral dose of deuterium oxide (stable isotope-tracer) provided to the lactating mother. Thus, through BM, the tracer is transferred from mother to child, allowing the quantification of BM volume consumed by the infant. Additionally, the method discriminates between exclusive and partial breastfeeding and does not require strict schedules for BM sampling, allowing a more natural study of BM intake without interfering with the normal activities of the mother-infant pair.

Considering the above, in order to reduce VAD in nursing infants, we need a further understanding of the association between the VA status of the mother and its impact on the breastfed infant. Therefore, we aimed to combine two methods (i.e., dose-to-mother DOD and BM retinol concentration based on volume and fat content), to assess the VA status of lactating women, estimate the transfer of retinol from the mother to the breastfed child, and evaluate the nutritional adequacy of VA in breastfed children from an urban and an agricultural area.

## 2. Subjects and Methods

### 2.1. Study Design and Bioethical Considerations

This was a cross-sectional design where breastfed infants and their mothers were selected by invitation from one of two study areas, i.e., urban and agricultural areas, in Sonora, Mexico. The study was approved by the Bioethics and Research Committee from the Department of Medicine and Health Sciences of University of Sonora (DCMS/CBIDMCS/D-23 20 March 2012; addendum for BM retinol analysis: DMCS/CBIDMCS/D-37 12 February 2013) endorsed by the Mexican National Bioethics Commission. Written informed consent was obtained from participants.

The study areas included Hermosillo, the capital city of Sonora state, as the urban area and Poblado Miguel Aleman, located in the Sonoran Coast, as the agricultural area. Participants who declared exclusive breastfeeding (EBF) or predominant breastfeeding (PBF) were recruited from local health clinics participating in health counseling programs during October 2012–March 2013.

Sample size was calculated based on a theoretical BM volume difference of 100 mL between EBF and PBF women and a standard deviation of 130 mL/day with an alpha level of 0.05 and a beta of 0.80 [18]. Thus, the calculated sample size was 30 mother-infant pairs from each area.

### 2.2. Selection Criteria

Adult mothers who declared EBF or PBF practices and resided for more than a year in one of the two study areas, were eligible to participate in the study. Additionally, their infants had to be born at full-term pregnancy (38–42 weeks gestation) and 3–6 months old at recruitment. Exclusion criteria included multiple births or infants in the weaning process, as well as mothers that consumed alcohol, tobacco, or drugs. Mothers who failed to complete the ‘dose-to-mother’ protocol and infants whose quantified BM intake was below 250 mL/day, were excluded because in a well-established breastfeeding process the infant’s BM intake is typically between 620 and 660 mL/day in developing countries [19].

### 2.3. Anthropometry

Anthropometric measurements were performed as described by Cameron, 2004 [20]. Mothers’ body weight and height were measured using an electronic scale (SECA^®^-876, Seca gmbh & Co., Hamburg, Germany) and a portable stadiometer (SECA^®^-217, Seca gmbh & Co.), respectively. From both measurements, body mass index (BMI) was calculated and classified according to WHO criteria. Infants’ body weight was measured with a pediatric electronic scale (SECA^®^-728, Seca gmbh & Co.) and length using an infantometer (SECA^®^-416, Seca gmbh & Co.). Infants’ anthropometric indices, i.e., weight for age, length for age and weight for length, were classified according to WHO growth reference *Z*-scores, using the software Anthro v 3.2.2 (World Health Organization, Geneva, Switzerland) [21].

### 2.4. Deuterium Oxide Dilution ‘Dose-to-Mother’ Technique

After anthropometric measurements, basal saliva samples from both mother and infant (5 mL and 2 mL, respectively) were collected and placed in cryogenic vials, in order to determine the deuterium natural abundance in participant mother-infant pairs. Next, an oral dose of deuterium oxide (deuterium-labeled water) was provided to the mother (~30 g; Cambridge Isotopes Laboratories, Andover, MA, USA). Deuterium oxide was allowed to mix in the mother’s total body water pool, during which it was naturally transferred to the breastfed infant through BM. Mother-infant saliva samples were collected on days 1, 2, 3, 4, 13 and 14 post-dose. All saliva samples were stored at −20 °C until their analyses at the Nutrition and Body Composition Laboratory (University of Sonora).

BM and other liquids consumed by the infant, were calculated according to a water turnover model using deuterium-oxide as a tracer and water as the tracee [17]. Mother’s isotopic enrichment followed a single exponential equation:

(1) $$E_{m\left(t\right)}=\;E_{m\left(0\right)}e^{-kmmt}$$

where *E_m(t)_* refers to the isotopic enrichment in the mother’s body at time *t* (ppm), *E_m(0)_* is the enrichment at time zero (ppm), t is the time post-dose saliva sample were taken and kmm is the water turnover in the mother (1/day).

For each infant, data were fitted as a double exponential based on the fact that infants could receive liquids from BM and non-milk sources. The infant’s equation is:

(2) $$E_{b\left(t\right)}=E_{m\left(0\right)}\left(\frac{F_{bm}}{V_{b}}\right)\left(\frac{e^{-k_{mm}t}-e^{-\left(F_{bb}/V_{b}\right)t}}{(F_{bb}/V_{b})-k_{mm}}\right)$$

where *E_b(t)_* refers to the isotopic enrichment in the infant’s body at time *t* (ppm), *F_bm_* is the water transferred from the mother to the infant through BM (kg/day), *V_b_* is the infant’s total deuterium distribution (kg) and *F_bb_* is the total water loss in the infant (kg/day).

Parameters fitted are *E_m(0)_*, *F_bm_*, *K_mm_* and *F_bb_*. *V_b_* is assumed to change in a linear manner with weight (W, kg) during the experimental period and was related to infant’s W as: *V_b_* = 0.84 W^0.82^ [18].

The deuterium enrichment of the saliva samples was determined by Fourier transform infrared spectrometry (Nicolet iS 10, ThermoScientific, Waltham, MA, USA). BM and non-milk intake were calculated by fitting the deuterium enrichment data to a model of water turnover in the mother and the infant using the Solver function of Microsoft Excel^®^ based on the principles of these equations, proposed by Coward et al. 1982 [17] and validated by the International Atomic Energy Agency (IAEA). Another advantage of using the DOD technique is that at the same time one can measure body composition of the mother (fat-free mass and fat mass) [18].

Infant feeding patterns were classified according to WHO [22] whereby EBF infants receive only BM from their mother and no other liquids or solids with the exception of drops or syrups consisting of vitamins, mineral supplements, or medicines.

### 2.5. Breast Milk Sample

A casual sample of BM was collected, i.e., a small amount of BM at no particular time since the last breastfeeding episode. This method is a recommended biochemical indicator by WHO to assess VA status in lactating women and it is further supported by other studies [23,24].

BM collection was performed by stimulation using a manual breast pump around 11:30 a.m. (±2 h). After five seconds of milk production, BM samples were collected and then placed in amber glass vials (5 mL). Subsequently, fresh BM samples were placed on frozen gel packs and stored in an icebox, protected from light, and transported to the Nutrition and Body Composition Laboratory (University of Sonora), where they were stored at −20 °C until BM retinol and carotenoid concentrations were analyzed by HPLC.

In BM, VA is found primarily in the milk fat globules, thus alterations in BM fat content also affect BM retinol and carotenoid concentrations. Therefore, previous to HPLC analysis, BM samples were thawed, homogenized, and milk fat percentage was calculated using the creamatocrit method described by Lucas et al., 1978 [25].

### 2.6. Breast Milk Retinol and Carotenoid Concentrations

BM VA content was determined using HPLC after an aliquot (500 µL) of milk was saponified. Briefly, 750 µL ethanol was added and the vial was mixed by vortex 15 s, then 400 µL KOH:HOH (50:50, w:v) was added and mixed 15 s, and placed in a water bath at 45 °C for 60 min, mixing the vials every 15 min [10]. Later the sample was extracted twice with 1 mL hexanes, mixed for 30 s, and centrifuged 10 min at 4 °C and 3000 rpm. The upper hexane layer was collected and placed in a test tube for subsequent evaporation under a gentle stream of nitrogen. The residue was reconstituted in 100 µL ethanol and injected onto the HPLC system using a C30 YMC S-3-Carotenoid (3 µm, 150 × 4.6 mm) column and the method described by Yeum et al., 1996 [26]. The system was an HPLC Agilent-1220 equipped with a photodiode array detector (Infinity 1260, Agilent Technologies, Santa Clara, SA, USA).

For quality control we used echinenone as an internal standard. HPLC-purified standards were used to calculate retinol, lutein, zeaxanthin, β-cryptoxanthin, α-carotene, all-*trans*-β-carotene, and lycopene concentrations by using an external calibration curve obtained from standards (Sigma-Aldrich, St. Louis, MO, USA). The inter-run variation was 9% for retinol and 15% for total carotenoids (based on the analysis of pooled BM sample).

BM retinol and provitamin A carotenoid concentrations were expressed by volume (µmol/L and nmol/L, respectively), and retinol was also expressed per gram of milk fat (µg retinol/g milk fat). According to WHO, in lactating women VAD is defined when BM retinol concentration is <1.05 µmol/L or ≤8 µg retinol/g milk fat [1].

### 2.7. Adequacy of Infants’ Vitamin A Intake

To assess whether consumption of VA in participant infants was adequate, we considered the volume of milk that was consumed per day and the BM retinol concentration by volume. Additionally, we considered the contribution of BM provitamin A carotenoids to the infant’s VA dietary intake using the conversion factors recommended by the Institute of Medicine-Food and Nutrition Board (i.e., 12 µg β-carotene and 24 µg β-cryptoxanthin and α-carotene per 1 µg of retinol). Breastfed infant’s VA intake was then compared with the recommended dietary adequate VA intake (i.e., 400 retinol activity equivalents (RAE)/day) [27].

### 2.8. Statistical Analysis

Descriptive statistics were generated. Values in the text are means ± standard deviations. Analysis included proportions for categorical variables and measures of central tendency for continuous variables. Differences between groups were examined by χ^2^ test, Student *t*-test or ANCOVA for parametric variables, and Mann-Whitney *U* or Wilcoxon test for nonparametric variables. For continuous variables, tests were performed assuming a 2-tailed analysis and differences were considered significant at *p* < 0.05. Correlations between variables of interest were assessed by Pearson’s correlation coefficient. Data analysis was performed using the NCSS statistical software (NCSS 2016^®^, Kaysville, UT, USA, v 11.0.6).

## 3. Results

Fifty-nine lactating women were enrolled, but 3 were excluded from the analysis because BM intake by the infants was <250 mL/day. Analyzed data include the 56 lactating women-infant pairs that met the criteria for participation (26 and 30 from the urban and agricultural areas, respectively). Table 1 shows the general characteristics of the mother-infant pairs.

When assessing lactating women’s BMI individually, 5.4% (*n* = 3), 41% (*n* = 23) and 53.6% (*n* = 30) were classified as low weight, normal, and overweight, respectively, and no difference was observed between areas (*p* = 0.16). There was a positive association between fat mass percentage and BMI in participant lactating mothers (*p* < 0.01), and 60.7% (*n* = 34) of them showed high levels of body fat, when contrasted with the reference values for non-pregnant and lactating women [28].

Even when the average for all nutritional indicators of participant infants were normal, based on the individual analysis of the Z scores of anthropometric indicators, 6 infants were below −2 Z scores of weight/length (2 and 4 from the urban and agricultural areas, respectively) and 1 was below −2 Z scores of height/age (urban).

BM retinol and carotenoid concentrations are shown in Table 2. Mean BM retinol concentration was 1.1 µmol/L, and when comparing to the cut-off point for deficiency (i.e., BM retinol <1.05 µmol/L), 57% (*n* = 32) of participant women had VAD. Even though BM retinol concentration (by volume) was significantly lower in the agricultural area (*p* = 0.028), the prevalence of VAD was similar in both areas, 60% (*n* = 18) and 54% (*n* = 14), for the urban and agricultural areas, respectively (*p* = 0.64).

Fat is an important factor to account for when assessing retinol and carotenoid concentrations in BM. In our study population, BM fat concentration was 23 ± 14 g/L and no difference was observed between areas (*p* = 0.43). Calculating the VA concentration (µg retinol) per gram of milk fat as an alternative indicator of VA status for lactating women, VAD was identified in only 16% (*n* = 9) (*p* = 0.15). We analyzed the association between body composition categorized by BMI (<18: thinnest; 18–24.9: normal and ≥25: overweight) and BM retinol concentration (by volume and per gram of milk fat), and there was no statistical difference (*p* > 0.05 for both variables) (Table 3). Although, when analyzing the relationship of stunting in early life of participant mothers on their VA status, stunted women (*n* = 14) tended to have lower BM retinol concentration (*p* = 0.058) than those with normal height.

The major provitamin A carotenoid found in BM was β-carotene (95%), followed by β-cryptoxanthin (4%) and α-carotene (1%). When considering total carotenoids in BM, β-carotene was still the most abundant (50%), followed by lutein (34%), zeaxanthin (10%), β-cryptoxanthin (4%), α-carotene (1%), and lycopene (1%). There was a positive and significant association between BM retinol and provitamin A carotenoid concentrations: α-carotene (*r* = 0.61; *p* < 0.001), β-carotene (*r* = 0.58; *p* < 0.001) and β-cryptoxanthin (*r* = 0.43; *p* < 0.001).

Table 4 shows the BM intake by the infants as well as their VA dietary adequacy, either from preformed VA (retinol) or provitamin A carotenoids in addition to preformed VA. BM intake tended to be lower in infants from the urban area (*p* = 0.067). Given the precision of the dose-to-mother method we were able to identify EBF (non-BM fluid intake 71 ± 56 g/day) in 62.5% (*n* = 35) and PBF (non-BM fluid intake 427 ± 176 g/day) in 37.5% (*n* = 21) of participant lactating mothers. The BM intake tended to be higher for EBF infants (777 mL) vs those PBF (599 mL) (*p* = 0.05), also there was no statistical difference in BM retinol concentration between EBF and PBF (either by volume or per gram of milk fat). Additionally, by comparing the dose-to-mother results with the mother’s declaration of type of breastfeeding, it was possible to observe that 70% (*n* = 39) accurately described if they were EBF or PBF.

According to the intake of BM by the infants and the amount of retinol and provitamin A carotenoids in BM, we observed that infants from the agricultural area tended to have a lower VA adequacy compared with those from the urban area (*p* = 0.054). On average, infants only consumed 229 out of 400 RAE/day (either quantified by volume or per gram of fat), which is the adequate intake for this age group. Taking into account the above, failure to meet the daily VA recommended intake was observed in 77% (*n* = 20) and 97% (*n* = 29) of breastfed infants from the urban and agricultural areas, respectively. Considering the mother’s nutritional status, infants from stunted mothers met only 41% of their daily recommended intake vs. 63% on infants of women with normal height (*p* = 0.029).

After considering provitamin A carotenoids as part of the daily dietary VA intake of participant breastfed infants, a small but significant increase was observed (231 RAE; *p* < 0.001); and this led to a reduction in VA inadequacy from 88% to 86%. As expected, we found a positive and significant association between total VA intake and the amount of fat in BM (*r* = 0.43; *p* = 0.001).

## 4. Discussion

This study combined two methodologies (i.e., dose-to-mother DOD and BM retinol concentration), which allowed us to assess the VA status of the mother, as well as the VA intake of the breastfed infant. An advantage of our study design lies in the reduction of experimental bias, because the DOD technique quantifies an accurate amount of BM consumed by the breastfed infant and does not rely on weighing the infant before and after a feed. Additionally, the dose-to-mother method discriminates between EBF and PBF. As seen in our results, only 70% of participant women accurately self-reported EBF or PBF, this may represent bias when interpreting results from studies that rely only on self-report breastfeeding practices.

The theoretical consumption of milk used by other authors is between 600 mL/day [29] and 800 mL/day [30]. Furthermore, the US Institute of Medicine established the daily adequate intake of VA for breastfed infants based on a theoretical BM consumption of 780 mL/day. The BM intake by enrolled infants was within the range mentioned above and consistent with data published by other authors who have used the ‘dose-to-mother’ technique, 759 ± 142 mL/day [31] and 637 ± 247 mL/day [32], to quantify consumption of BM by the infant. On the other hand, we have the limitation of the collection of a casual sample of BM which may not reflect the diurnal variations in BM fat [33,34] and this may affect the concentration of VA. Nonetheless, this is a recommended biochemical indicator by WHO and has been supported by other studies [23,24]. Furthermore, we standardized the time of collection to be at 11:30 a.m. ± 2 h to reduce variation.

According to WHO, the percentage of VAD observed among participant lactating women using BM retinol as an indicator, represents a problem of public health concern [1]. Fifty seven percent of women showed low BM retinol (µmol/L), which drops to 16% when corrected for BM fat, confirming other findings that list Mexico as a country where VAD is a public health problem in different population groups [1]. Regardless of adjustments, VA intake of these infants is still below the recommended dietary intake [27]. Correction for fat proved to be important in this study, but logistically it is often cumbersome to determine in population surveys. This is especially true when the creamatocrit and volume aliquots cannot be performed on fresh milk, which then requires re-homogenization before analysis in the laboratory [10]. Additionally, at a national level often rural areas are most affected by VAD [5], which was also observed in our results, where the women of the agricultural area had significantly lower concentrations of BM retinol, when compared with those of the urban area (*p* < 0.05). Recent findings in Thailand show that BM retinol in hindmilk, which typically has higher fat content, is strongly associated with total body VA stores in lactating women (Tanumihardjo, personal communication) [35]. Even though hindmilk was not specifically collected in our study, the prevalence of low BM retinol is high and we can infer that participant lactating women’s VA stores may be depleted.

All infants are born with low stores of VA and BM is the infant’s main source of VA to meet requirements and build liver stores [36], but in areas where VAD is common, concentrations of BM retinol are observed to be 1 μmol/L [3], similar to our study. This amount only meets the metabolic needs of the baby and does not allow accumulation of liver stores of this nutrient [4]. Furthermore, our results suggest that the mother’s nutritional status (i.e., malnutrition in early life) was a limiting factor for VA adequacy of the breastfed infant (*p* = 0.029), and thus should be a variable to consider when assessing the VA status of lactating women.

A trend towards increased consumption of BM for infants from the agricultural area occurred (*p* = 0.067), perhaps as a compensatory mechanism to receive a greater amount of nutrients, given that no difference was found between areas when using BM retinol concentration by gram of fat (*p* = 0.438). There is an assumption that BM from well-nourished mothers in developing countries has an average of 38 g/L of fat, when 24 h BM collection is performed [37,38]. We compared our results with a calculated estimate of vitamin A intake assuming that BM fat was a constant of 38 g/L for all participant women, and the result (i.e., 404 µg VA/day) almost doubles our quantified VA intake per gram of BM fat (*p* = 0.000; 95 CI 314–504 µg/day). Additionally, inadequacy of VA intake was reduced to 50% vs. 88% found in our study. Hence, this reiterates the importance of the dose-to-mother method, and when possible, individual determinations of BM fat concentration in the field to accurately estimate BM volume and adequacy of fat-soluble micronutrient intakes by the breastfed child. Although BM fat analysis is logistically difficult in population studies, using a fixed concentration of BM fat can mask nutritional deficiencies, especially in developing countries where mothers may not have an adequate nutritional status.

When assessing the VA adequacy of breastfed infants, the Institute of Medicine established the daily adequate intake based on the consumption of 780 mL BM/day by the infant. We compared our results (i.e., quantified BM intake) with a calculated estimate of VA intake assuming only 780 mL BM/day and no statistical difference was found (*p* > 0.05), perhaps because the BM intake of our infants was close to the Institute of Medicine’s assumption. Additionally, the Institute of Medicine does not consider the contribution of provitamin A carotenoids to meet the infants VA requirement, because the bioconversion of carotenoids from milk in infants is not known [27]. Nonetheless, we explored the impact of including the carotenoid intake from BM on the VA dietary adequacy of the infants and it showed a significant increase in VA intake (*p* < 0.001). We also used a high bioconversion factor, i.e., 12 μg β-carotene equivalents to 1 μg retinol, which likely underrepresents the actual bioconversion rate in infants with VAD.

Total carotenoid concentration observed in this study (105 nmol/L) considerably differs from a previous study on carotenoid content in BM of Mexican well-nourished lactating women (223 nmol/L) but is similar to that observed in women from the US (114 nmol/L) [39]. This can be explained by the similarities in dietary habits of the northwest region of Mexico and the US. The carotenoid profile found in this study differs from what Canfield et al. [39], described for Mexican lactating women where β-cryptoxanthin was the most abundant carotenoid, but is similar to a more recent report by Lipkie et al. 2015 [40] in Mexican lactating women where β-carotene was the most abundant.

An additional advantage of the ‘dose-to-mother’ technique is the simultaneous assessment of the lactating mother’s body composition. At present, cut-off points to define excess or limited body fat percentage in lactating women are not available, but when considering body fat combined with a BMI ≥ 25 more than 50% of participant women were overweight or obese. Nevertheless, the physiological rise in fat stores during pregnancy supports lactating women in BM production given that EBF women require over 640 kcal in addition to their pre-pregnancy energy requirement [41]. Even when there are not enough data to estimate the amount of body fat lactating women must have during lactation, our study showed that their fat stores were sufficient to sustain an average of 758 mL/day of BM production. On the other hand, we found that 25% (*n* = 14) of the women had low height (<150 cm) [42], which is a sign of malnutrition in early life, and these women tended to have lower BM retinol concentrations when compared to those of normal stature (*p* = 0.058).

By combining the ‘dose-to-mother’ method with the determination of BM retinol, it was possible to demonstrate VAD in the population and quantify the amount of VA that the infants received while breast-feeding. Although serum retinol concentrations were not determined directly in the infants, which is a limitation of this study, BM retinol concentrations combined with BM volume suggested low VA intakes.

## 5. Conclusions

BM is the only source of food for EBF infants, and because of the high prevalence of VAD among Mexican lactating women, strategies to reduce VAD in the population must target both children under 5 years as well as lactating women, in order to increase the chances of improving VA status in both groups. The use of nuclear techniques (stable isotopes) in vulnerable populations, particularly in lactating women and infants, such as the dose-to-mother DOD and stable retinol isotope dilution, could improve status assessment of VA and provide a better and more accurate estimate of VA dietary requirements for breastfed infants.

## Figures and Tables

**Table 1:** General characteristics of participant mothers and infants.

	Total (*n* = 56)	Urban Area (*n* = 26)	Agricultural Area (*n* = 30)	*p*
	Mean ± Standard Deviation			
***Mothers***				
**Age (Years)**	25 ± 7	27.8 ± 6	22.4 ± 7	0.003
**Weight (kg)**	62 ± 12.8	63 ± 11.5	70 ± 14	0.49
**Height (cm)**	157 ± 8.6	161 ± 6.3	153 ± 8.7	0.000
**BMI**	25 ± 4.8	24.5 ± 4	26 ± 5	0.26
***Infants***				
**Age (months)**	3 ± 2	3.6 ±1.5	3.1 ± 1.6	0.24
**Weight (kg)**	6 ± 1	6.4 ± 0.9	6.1 ± 1.4	0.30
**Length (cm)**	61 ± 4.5	61.1 ± 3.7	60 ± 5	0.51
**Weight for age (*Z* score)**	0.1 ± 1.2	0.1 ± 1.0	0 ± 1.3	0.75
**Length for age (*Z* score)**	−0.1 ± 1.5	−0.2 ± 1.3	0 ± 1.6	0.65
**Weight for length (*Z* score)**	0.3 ± 1.4	0.5 ± 1.4	0.2 ± 1.4	0.51

**Table 2:** Concentration of retinol and carotenoids found in breast milk of participant lactating mothers.

	Total	Urban Area (*n* = 26)	Agricultural Area (*n* = 30)	*p*
	Mean ± Standard Deviation			
**Retinol µmol/L**	1.1 ± 0.6	1.3 ± 0.69	0.92 ± 0.51	0.028
**Retinol µg/g milk fat**	16 ± 8.4	16.3 ± 8.7	15.7 ± 8.3	0.799
**Lutein (nmol/L)**	30.7 ± 21.2	36 ± 21.6	26 ± 20	0.083
**Zeaxanthin (nmol/L)**	7.7 ± 4.4	8.9 ± 5.2	6.8 ± 3.4	0.073
**β-cryptoxanthin (nmol/L)**	2.5 ± 3.2	4 ± 4.2	1.2 ± 0.7	0.001
**α-carotene (nmol/L)**	0.89 ± 1.1	1.5 ± 1.3	0.38 ± 0.29	0.000
**β-carotene (nmol/L)**	62.2 ± 80.7	100 ± 105	29.7 ± 22.5	0.001
**Lycopene (nmol/L)**	1.1 ± 0.9	1.5 ± 1.2	0.73 ± 0.60	0.002
**Provitamin A carotenoids (nmol/L)**	65.7 ± 84.5	105 ± 110	31.2 ± 23.2	0.001
**Total BM carotenoids (nmol/L)**	105 ± 104	152 ± 133	64.8 ± 40.1	0.001

**Table 3:** Vitamin A concentration (by volume or per gram of milk fat) and fat mass percentage categorized by body mass index (BMI) (<18: thinnest; 18–24.9: normal and ≥25: overweight).

BMI	% Fat Mass	VA µmol/L	VA µg/g Fat
	Mean ± Standard Deviation		
<18 (*n* = 3)	24 ± 0.4 ^a^	0.74 ± 0.2	10 ± 1.7
18–24.9 (*n* = 23)	29 ± 8.6 ^a^	1.06 ± 0.6	16 ± 8
≥25 (*n* = 30)	38 ± 5.4 ^b^	1.15 ± 0.6	16 ± 9
*p*	0.000	0.533	0.491

^a,b^ Different letter in column means statistical difference.

**Table 4:** Breast milk intake and adequacy of intake of vitamin A (VA) in participating infants.

	Total (*n* = 59)	Urban Area (*n* = 26)	Agricultural Area (*n* = 30)	*p*
	Mean ± Standard Deviation			
**Breast milk intake (mL)**	758 ± 185	710 ± 169	800 ± 190	0.067
**Retinol intake (µg/day)**	229 ± 129	265 ± 155	197 ± 93.2	0.048
**Adjusted retinol intake per g of BM fat (µg/day) ^1^**	229 ± 129	259 ± 23 *	202 ± 21 *	0.072
**VA ^2^ intake (RAE/day)**	231 ± 130	268 ± 157	198 ± 93.6	0.043
**Mean Adequacy of VA intake (%)**	57 ± 32	66 ± 39	49 ± 23	0.054

^1^ BM retinol intake for agricultural and urban areas was adjusted using BM fat concentration [25] as a covariable. * Values are presented as adjusted means and standard error. ^2^ VA: includes retinol and provitamin A carotenoids (α- and β-carotene and β-cryptoxanthin).
